# Analysis of Radon‐222 in Surface and Groundwater Sources in Kajiado County, Kenya

**DOI:** 10.1155/tswj/6345685

**Published:** 2026-03-03

**Authors:** Christine Kerubo Onyoni, Calford Otieno, Jeremiah Monari Kebwaro

**Affiliations:** ^1^ Department of Physics, Kisii University, Kisii, Kenya, kisiiuniversity.ac.ke; ^2^ School of Pure and Applied Sciences, Karatina University, Karatina, Kenya, karu.ac.ke

**Keywords:** groundwater, radon concentration, surface water

## Abstract

This study entailed measurement of the concentration of ^222^Rn in surface and subsurface water sources from Kajiado Municipality in Kenya. Kajiado County receives minimal rainfall and is not well covered by the municipal treated water network. The residents of the area mainly rely on subsurface water sources for their domestic consumption. Twenty‐seven surface and groundwater samples were collected, and ^222^Rn concentrations were measured using a RAD7 alpha detector. It was observed that the radon activity in groundwater ranged from 0.62 to 13 mBq/L while the radon activity in surface water ranged between 0.06 and 1.53 mBq/L. The mean values obtained were 6.01 ± 1 mBq/L for the groundwater and 0.45 ± 0.11 mBq/L for the surface water. These values are within the US Environmental Protection Agency (USEPA) standard limit of 148 mBq/L and the World Health Organization (WHO) global average of 100 mBq/L. The water is generally safe for domestic consumption.

## 1. Introduction

Human beings have always been exposed to ionising radiations of natural origin. Natural sources of radiation include emission from the decay chains of ^232^Th and ^238^U which occur naturally in geological materials.

Public concern over radiation exposure emphasises artificial radiation sources, especially from nuclear facilities. However, the greatest exposure to the population is caused by natural radiation sources. The growing worldwide interest in natural radiation exposure has led to extensive surveys in many countries [1]. Estimation of natural radioactivity is therefore necessary in evolution of the dose rate for outdoor occupation [2]. Radon (^222^Rn) is a naturally occurring radioactive gas that is common in many human dwellings including homes and workplaces. It is considered to be a major risk factor for lung cancer in the never‐smoking population worldwide and is reported as the second major cause of lung cancer after tobacco smoking [3]. The gas is produced from the natural radioactive decay of uranium, found in rocks and soil [4]. Radon escapes from the ground into the air, where it decays to daughter radioactive isotopes. The daughter radioactive products are either short‐lived (^218^Po, ^214^Pb, ^214^Bi and ^214^Po) or long‐lived (^210^Pb, ^210^Bi and ^210^Po). As we breathe, the solid decay products are deposited on the lung cells, where they can damage DNA and cause lung cancer. Most of the radiation hazards of radon are due to its progenies, which emit alpha particles, and are considered very ionising when interacting with living cells [5]. An important secondary source of radon exposure is groundwater which is stored in the houses for domestic use. Water collected from boreholes or wells sunk in areas with elevated natural radioactivity would contain high levels of radon gas. When this water is stored in human dwellings, the dissolved radon continuously seeps into the house, hence significantly contributing to indoor radon in the said houses and further raising the rate of exposure [6]. Currently, Kenya does not have a policy framework for the assessment of radon levels in water sources and the establishment of regulatory limits for portable water. This study investigated the concentration of ^222^Rn in surface and subsurface water samples from Kajiado County in Kenya. Kajiado County is a semiarid with intermittent rainfall, hence persistent water scarcity in the area. Most residents rely on subsurface water from shallow wells, boreholes and stagnant surface water for domestic consumption. The radon concentration in these sources is not documented, nor are there legal instruments on water quality for subsurface sources in private residences. The study presents useful information to guide policy on water resource management and radiation safety. Globally, research shows that radon concentrations in drinking water can vary widely. To provide an example, the radon content in groundwater established by a study in the United States ranged up to 150 Bq/m^3^ [7], and this content translates to far greater ingestion doses than the doses that were found in this study. The same studies have shown that mean levels of radon in surface waters in Europe tend to be less than 10 Bq/m^3^, causing reduced risks to health [8]. Ismail et al. recorded the evaluation of the ^222^Rn concentration of groundwater in the south‐western part of Malaysia (Malacca). The present study collected 27 samples and was able to measure the ^222^Rn concentration at ^222^Rn using the RAD7 handheld ^222^Rn detector and measure ^222^Rn concentrations on 27 samples. The observed ^222^Rn levels were in the parameters of 7.4–89.1 Bq/L, and this is lower than the reference limit of 100 Bq/L recommended by the World Health Organization, as well as the radiological risk which ranged between 0.02 and 39.2 *μ*Sv/y with the average of 4.5 *μ*Sv/y [9].

The importance of the dose conversion factor (DCF) cannot be overestimated when it comes to the translation of the radon activity in effective doses, which allows one to compare them across different water sources. Although the value of DCF applied in this paper is small, higher doses of radionuclides ingested are largely determined by the increased levels of radon present in the sources such as the earth dam. That creates the urgency of specific water treatment and mitigation measures, particularly in the geologically rich areas that would ensure the minimisation of health risks posed by radon ingestion on the local and global scales.

## 2. Study Area

**Figure 1:**
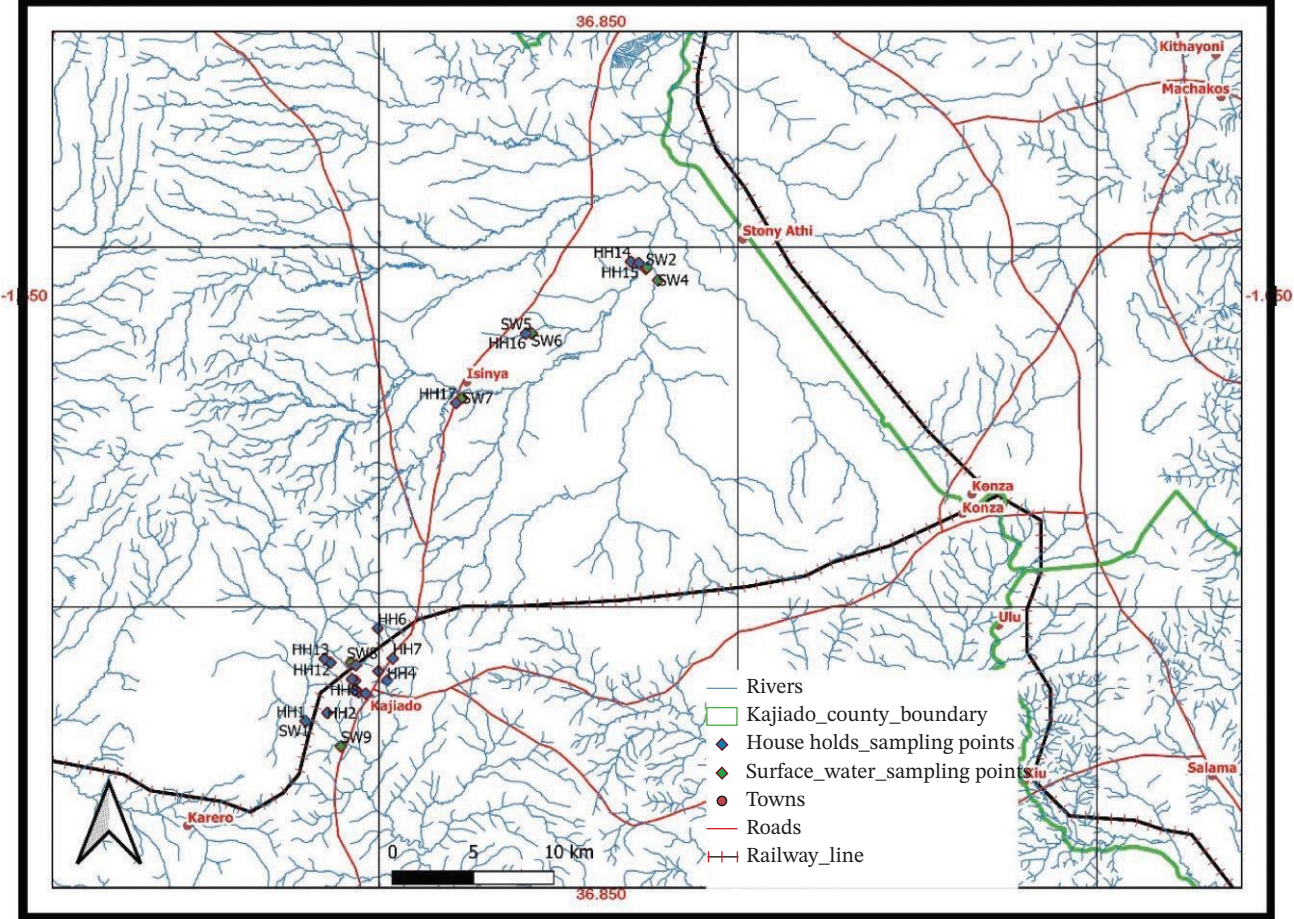
Sampling map and profile.

The research was conducted in Kajiado County, which lies along the Nairobi–Arusha highway, south of Nairobi. The area is surrounded by several water sources, including rivers, earth dams and springs. The most common among the rivers are the Athi River, Olkejuado River and Isinya River. However, many of the residents get their drinking water and that for domestic use from the subsurface sources. Figure 1 shows the map of the study area and the points where sampling and measurement of radon in water were conducted.

### 2.1. Rainfall Pattern and the General Hydrology of Kajiado

It is estimated that the average annual rainfall in the area of Kajiado Town is 400 mm/year. Rainfall is biannual; that is, short rains occur between the period October and December and long rains between March and May. The rainfall distributions are shown in Figure 2. The average area has a high incidence of evapotranspiration, owing to hot weather. There is an excess of precipitation during the short and long rains compared to the actual evapotranspiration (M, I, & H, 2019).

Figure 2The (a) annual precipitation and (b) monthly mean precipitation variability for Kajiado (Oord, 2017).

**Figure:**
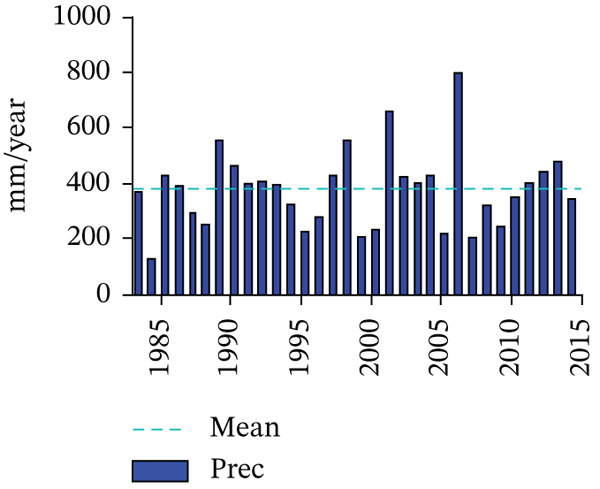
(a)

**Figure:**
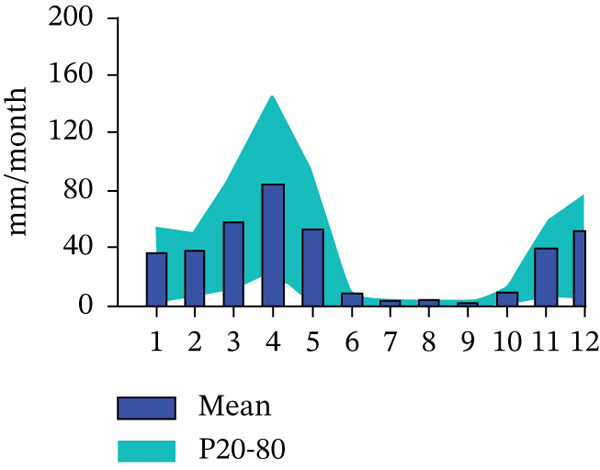
(b)

Kajiado Town technically is situated within the Athi River Basin. Athi River is located to the north of the county. It has a tributary river Kajiado River which is located 3 km west of town. Figure 1 shows Kajiado Town and its subcatchments. The Kajiado River headwater region falls on the northern and northwestern side of town and has a catchment area of 200 km^2^ up to the river head southeast of Kajiado. Kajiado Town is located in a water divide; the water flowing along the south of the main road (Namanga roads) is drained into the south and flows into the Kajiado River. To the west of the road, water drains northwards to a further upstream part of the Kajiado River through three parallel east–west oriented gullies (a, b and c) as depicted in the map above (Figure 1).

### 2.2. Sampling Procedure

The systematic random sampling method was used in the collection of water samples from the study area. A total of 27 samples were collected. These were from the surface water sites (rivers, streams and swamps), groundwater (boreholes and shallow wells) in Kajiado Municipality and its environs which roughly covered all the geological surface formations. Each sample was coded uniquely as GW for groundwater samples and SW for shallow water sites. The samples were collected during the dry period of the year which was noted to be in the months of July to September [10]. Samples from rivers were taken from stagnant or from flowing water [11]. When a sample was taken from stagnant water, the container was fully immersed in the water body to a depth of approximately 150 mm below the surface of the water and then closed under water when it is completely full and devoid of air bubbles. Samples were collected using 250 mL glass bottles from a total of 27 sites for radon analysis. To minimise adsorption of metals onto the walls of the sample container, the container was washed off with 10% nitric acid [12], thoroughly rinsed with distilled water, and finally with the sample water before the water was collected.

In case of flowing water, the mouth of the container was pointed in the direction from which the water is flowing; then, the bottle was immersed completely in the water to ensure all the air in the container is completely evacuated. When sampling from a tap, the water was first allowed to run for a few minutes to let water from the possibly stagnant section of the pipe flow to purge the air [13]. Thereafter, the water was filled in the sampling containers. These measures were to minimise losses of radon gas while sampling and before analysis [12, 14]. During sampling, the water flow was adjusted to avoid turbulence [15]. The container was completely filled with water and closed with airtight cap to prevent radon gas from escaping out of the container.

#### 2.2.1. Sample Analysis: Measurement of Radon Concentration

**Figure 3:**
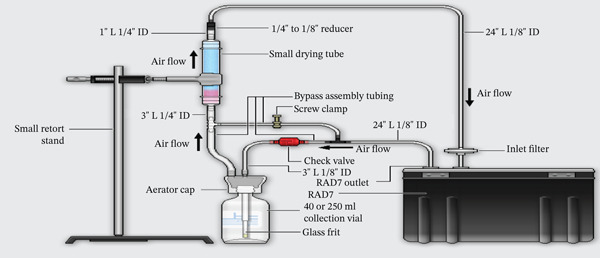
RAD‐H_2_O configuration.

Radon concentration in the water samples was determined in situ to avoid uncertainties that may be introduced by radioactive decay of radon‐222 [16]. The measurements were done using RAD7 Version 3 of 140129 Model 715 equipment from Durridge connected to the RAD‐H_2_O accessory with a 10% standard deviation (Figure 3). The RAD‐H_2_O technique employs closed loop concept, consisting of three components: the RAD7 with a radon monitor, the water vial with aerator and the tube of desiccant supported by the retort stand. The detector has a semiconductor material for converting alpha radiation to electrical energy.

The radon concentrations in water samples that were collected can be calculated using the following equation:

CRn=ASampleVSample XEfficiency,

where *C*_Rn_ is the radon concentration in the sample. *A*_sample_ is the activity of the radon in the sample, and *V*_sample_ is the volume of the water sample. Radon activity in this study was measured in picocuries per litre (pCi/L) and Becquerels per litre.

## 3. Results and Discussion

The first step in evaluating the radon‐222 levels in Kajiado Municipality was to determine the activity concentrations of this radioactive gas in both surface water and groundwater sources. These included the collection and analysis of samples of various sources such as rivers, springs, earth dam and house wells. Analysis of these varying concentrations of ^222^Rn in the various water sources would enable one to determine patterns and trends in the presence of the radon in the region in relation to the underlying geological and environmental factors affecting the distribution of radon. The following Table 1 indicates the different ^222^Rn concentrations in water at the points of sampling in Kajiado County.

**Table 1:** Measured ^222^Rn activity concentration in water at the sampling points in Kajiado County.

Sample no.	Name	Activity (mBq/L)
1	Household 1	1.406 ± 0.198
2	Household 2	0.618 ± 0.203
3	River	1.125 ± 0.277
4	Household 3	9.028 ± 2.689
5	Household 4	5.772 ± 1.428
6	Household 5	1.547 ± 0.093
7	Household 6	13.650 ± 3.248
8	Household 7	1.066 ± 0.119
9	Household 8	3.611 ± 1.180
10	Household 9	8.621 ± 2.327
11	Household 10	7.030 ± 0.384
12	Household 11	12.770 ± 3.344
13	Household 12	4.514 ± 0.328
14	Household 13	18.320 ± 4.625
15	Household 14	9.028 ± 2.689
16	Stoney Athi 1	0.164 ± 0.099
17	Stoney Athi 2	0.061 ± 0.046
18	Stoney Athi 3	0.262 ± 0.104
19	Household 15	6.253 ± 2.116
20	Household 16	3.097 ± 1.069
21	Spring Water 1	0.107 ± 0.026
22	Spring Water 2	0.237 ± 0.024
23	Isinya River	0.223 ± 0.063
24	Household 17	3.774 ± 1.328
25	Earth dam	1.513 ± 0.246
26	Household 18	1.043 ± 0.260
27	Okejuado River	0.340 ± 0.057

The activity levels of radon in this experiment ranged between 0.0611 mBq/L in Stoney Athi 2 and 18.32 mBq/L in Household 13, showing a significant difference. Radon levels in household wells were the highest with Household 6 and Household 11 recording high levels of 13.65 and 12.77 mBq/L, respectively. Conversely, surface waters including rivers and springs contained much smaller concentrations ranging below 1 mBq/L with the Stoney Athi River being low at all times. On the surface waters, the earth dam has a slightly higher level of 1.513 mBq/L. Such results suggest that the distribution of radon radiation in groundwater, particularly in domestic wells, is more erratic and probable to be affected by the local geology as well. The vast household sampling disparity makes predicting the existence of radon more difficult in support of the importance of localised radon measurements of water safety.

### 3.1. Analysis of ^222^Rn Concentrations in Surface Water Sources

**Figure 4:**
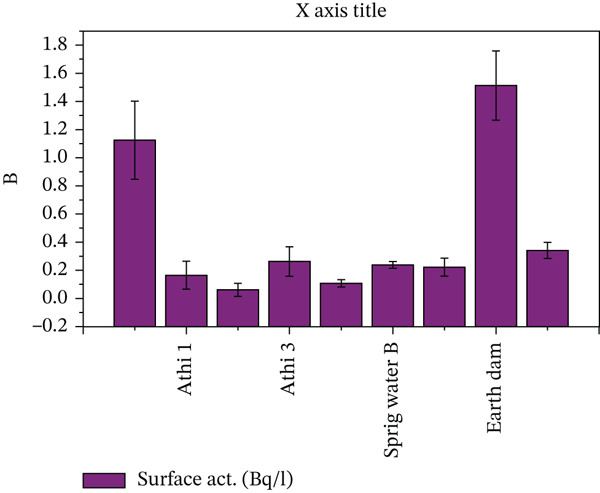
Presentation of ^222^Rn concentrations in surface water sources.

Figure 4 presents the analysis of ^222^Rn concentrations in surface water sources.

The Stoney Athi River samples (16, 17 and 18) exhibited relatively low concentrations of ^222^Rn, with levels ranging from 0.0611 to 0.2627 mBq/L. In contrast, River A sample (3) showed a slightly higher ^222^Rn concentration of 1.1248 mBq/L. Among the surface waters, the earth dam sample (25) displayed the highest concentration, measuring 1.5133 v. In the case of springs, Samples 21 and 22 revealed low to moderate concentrations of ^222^Rn, with values of 0.1073 and 0.2375 mBq/L, respectively. Similarly, the sample taken from an earth dam (Sample 25) stood out with a notably higher ^222^Rn concentration of 1.5133 mBq/L, significantly surpassing the levels found in the other surface water sources. It can be deduced; therefore, the generally lower concentrations in surface waters, particularly in rivers, can be attributed to the constant movement and aeration of water, which allows for the escape of radon gas to the atmosphere.

### 3.2. Analysis of ^222^Rn Concentration in Groundwater Sources

**Figure 5:**
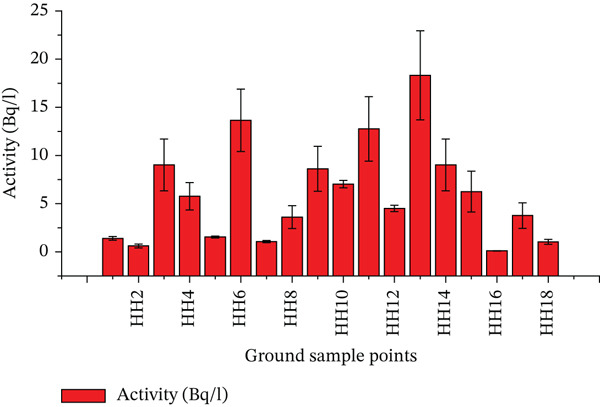
Presentation of ^222^Rn concentrations in groundwater sources.

Figure 5 presents values obtained from the groundwater sources.

Delving into the analysis of ^222^Rn concentrations in the groundwater sources, the study outcomes revealed a much wider range and generally higher levels of radon compared to the surface water samples. The groundwater sources, primarily consisting of household wells, displayed a diverse spectrum of ^222^Rn activity concentrations. On the lower side of the spectrum, there were two households characterised by rather low levels of radon. As can be seen, Household 2 (Sample 2) had a concentration of 0.6179 mBq/L, and Household 18 (Sample 26) had a concentration that is slightly higher but still low of 1.043 mBq/L. These households probably had geological conditions or properties of wells that were not favourable towards the build‐up of radon.

Conversely, there was a cluster of households with moderate concentrations of ^222^Rn; they indicated 1.066–3.611 mBq/L. This group included Households 1, 3, 5, 6 and 8 (Samples 1, 3, 6, 8 and 9). These moderate levels are not as high as those that follow but still should receive consideration and possible checking. The most worrying results were obtained through the high concentration households which had the levels of Rn ranging between 4.514 mBq/L up to the disgusting levels of 18.32 mBq/L. This group encompassed Households 4, 7, 10, 11, 12, 13, 14, 15, 16 and 17 (Samples 4, 5, 7, 10, 11, 12, 13, 14, 15, 19, 20 and 24). Household 13 (Sample 14) recorded the highest concentration of 18.32 mBq/L, which is an excellent reading as the concentration in other households was lower.

The great difference in the concentration of groundwater ^222^Rn can be explained by a number of factors. Potential contributors of the noticed differences are geological differences in the underlying bedrock, depth of the wells, residence of the water in the aquifer and presence of uranium bearing mineral in the local geology. These aspects lead to the mix‐up that causes the heterogeneous distribution of radon throughout the groundwater sources in Kajiado Municipality.

The comparison of such radon levels in groundwater offers useful information on the possible health threat that such concentration can cause to the native population and that there exists a necessity to develop specific monitoring and mitigation methods to guarantee the health and well‐being of the local population.

### 3.3. Comparison of ^222^Rn Concentrations in Surface Water and Groundwater Sources

The following examination of the ^222^Rn levels within the Kajiado Municipality indicates that there are some drastic variations in the surface water and the groundwater source. The range of ^222^Rn concentration in surface water samples was significantly smaller, as the range of values was between 0.0611 and 1.513 mBq/L. By contrast, the range of groundwater sources was much broader, fluctuating between 0.6179 and 18.32 mBq/L. It is worth noting that even the maximum amount of the pollutant, which occurred in surface water −1.513 mBq/L at the earth dam (Sample 25), was not reached by many groundwater samples.

The difference is further evident when a comparison is done between mean concentrations. The mean ^222^Rn concentration was much higher in groundwater sources due to the average of 5.7138 mBq/L when compared to the average 0.3761 mBq/L in the surface water sources. Such a sharp contrast implies that, by average, the concentration of ^222^Rn in groundwater within the study area is about 15 times higher than the concentration of the same element in surface water. Although the overall trend was more towards the increased concentrations in groundwater, there was a bit of overlap between the lower limit of the groundwater concentrations and the higher limit of the surface water concentrations. This overlap implies that there exist some caused factors of concentration that may build in common to both of these types of water origins.

**Figure 6:**
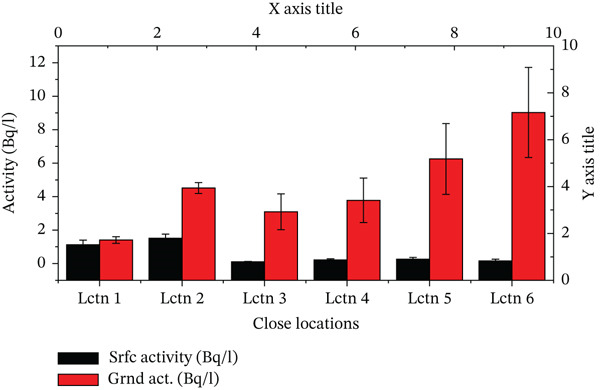
Graphs comparing the surface and the ground activities.

A visual comparison of radon activities in surface and groundwater is shown in Figure 6. It could empirically be stated and identified that the large disparity in ^222^Rn levels at the surface and groundwater sources is a strong positive indication that geological differences are critical. As it is in contact with the groundwater, which could have contained rocks that form 222Rn potentially, groundwater accumulates more radon in terms of ^222^Rn content. This geological impact is also supported by the fact that the variability in the concentrations of groundwater is greater, indicating that the local geological variation has a stronger impact on the concentration of radon in groundwaters.

### 3.4. Dose Assessment

The formula for calculating the annual effective dose is as follows:

AEDinh=14 CW×R×D×F×t.

Conversion factors that were used in the study were selected according to an already known guideline of reputable materials that provide the environment study on radiation safety. Constant *C*_*W*_ (0.1) was selected to normalise and standardise the dose calculations, which are commonly accepted in the environmental radon studies. The inhalation rate (*R*) of air (0.009 m^3^/h) represents an average breathing rate of individuals, based on generally used estimates of the adult breathing rate in usual surroundings. The DCF (*D*), which is 0.4, was obtained based on the literature and recommendations on radon to effective dose conversion, which is in line with the international standards provided by authorities such as the International Commission on Radiological Protection (ICRP). These considerations assure a reasonable level of consistency of the dose calculations consistent with the accepted practises and reliable evaluation of radon exposure risks in the studied environment [17]. The representative figures in Table 2 demonstrate the actual ingestion dose levels (microsieverts per year) of the water sources on the surface.

**Table 2:** Ingestion dose of ^222^Rn for surface water sources.

Surface activity (*μ*Bq/L)	Daily water consumption (L/day)	Dose conversion factor (DCF)	Time (days)	Ingestion dose (*μ*Sv/year)
1124.80	2	0.00000001	365	8.207
164.65	2	0.00000001	365	1.202
61.05	2	0.00000001	365	0.445
262.70	2	0.00000001	365	1.916
107.30	2	0.00000001	365	0.783
237.54	2	0.00000001	365	1.733
222.37	2	0.00000001	365	1.622
1513.30	2	0.00000001	365	11.040
340.77	2	0.00000001	365	2.484

The dose of radon‐222 (^222^Rn) in the surface water sources was estimated by multiplying the daily intake of water in litres per day, which is 1 L per day, with the conversion factor (DCF) of 1e − 8 (0.00000001) [13]. These doses ingested in the form of microsieverts per year give an understanding of the risk of exposure to the consumption of the water in such sources [12, 18, 19]. It is possible to obtain the ingestion dose through the formula: (Ingestion Dose = Surface Activity × DW × DCF × *T*).

Among the table and figures, one can distinguish the earth dam (where the activity is 1513.3 *μ*Bq/L) with the greatest ingestion dose of 11.04 *μ*Sv/year, which means that the overall health risk of the people who use this water source is significantly high. Comparatively, the doses of ingestion were very low in the Stoney Athi River samples, whereby the values were between 0.445 and 1.916 *μ*Sv/year. On the same note, spring water sources with other rivers registered a moderately low dose of 0.783 and 2.484 CSv/year.

The DCF plays a vital role in the process of converting the activity of radon into an effective dose, and thus, it allows comparing the water sources [20]. This aspect gives a crucial connection between the measured radiations of radon and the possible health effects of its consumption. Although the DCF values are low, with increasing levels of radon, the ingestion dose is greatly changed as has been observed in the earth dam, and thus, the localised water treatment or mitigation measures are required to limit the exposure. Table 3 indicates the calculated annual effective dose rate of the sources of groundwater (microsieverts per year).

**Table 3:** Calculation of annual effective dose rate for groundwater.

^222^Rn activity (mBq/m^3^)	DW	DCF	*T*	AED
1406	2	0.00000001	365	2.565
617.9	2	0.00000001	365	1.128
9028	2	0.00000001	365	1.648
5772	2	0.00000001	365	1.053
1546.6	2	0.00000001	365	2.822
13,653	2	0.00000001	365	2.492
1065.6	2	0.00000001	365	1.945
3611.2	2	0.00000001	365	6.591
8621	2	0.00000001	365	1.573
7030	2	0.00000001	365	1.283
12,765	2	0.00000001	365	2.329
4514	2	0.00000001	365	8.238
18,315	2	0.00000001	365	3.342
9028	2	0.00000001	365	1.647
6253	2	0.00000001	365	1.141
107.3	2	0.00000001	365	1.958
3774	2	0.00000001	365	6.888
1043.4	2	0.00000001	365	1.904

It can be observed that all samples fall below the WHO reference level of 100 Bq/m^3^, indicating that the water sources in Kajiado Municipality generally do not pose a severe radon‐related health risk. However, the variability in concentrations suggested the need for ongoing monitoring.

## 4. Conclusion

The study investigated radon concentration levels in the different water sources in Kajiado County. From the findings, it was generally observed that groundwater sources exuded higher radon concentration and activities compared to surface water sources. However, the majority of water sources showed relatively low levels of radon compared to the WHO reference level of 100 Bq/m^3^ for drinking water. This suggests that drinking water in Kajiado County is generally safe for human consumption. It is however recommended that water treatment strategies that would minimise radon levels be embraced before the water is consumed. The study recommends long‐term monitoring studies. This should be carried out in order to assess the concentration of the Radon‐222 gas at different periods of time. This will be useful in finding out the changes that may occur at different seasons and the changes that may occur over time as well as the effects of various environmental factors on the rate of production of Radon‐222. Also, this study would recommend detailed source characterisation whereby future researchers should examine the general geological and hydrogeological circumstances which have led to the build‐up of Radon‐222 in the water well. Further investigation of the degree and characteristics of the soils, the distribution of rock types and the pathways of percolating groundwater can yield information concerning the origins of Radon‐222. Lastly, the study recommends that there be effectiveness of mitigation techniques. More effort in research should be directed on effectiveness of various treatment processes as well as the cost implications and feasibility in the residential as well as in the municipal water treatment systems.

## Author Contributions

Christine Kerubo Onyoni: conceptualization, methodology, investigation and writing—original draft. Calford Otieno: supervision, validation and review. Jeremiah Monari Kebwaro: data curation, review and editing.

## Funding

No funding was received for this manuscript

## Disclosure

All authors have read and approved the final manuscript.

## Conflicts of Interest

The authors declare no conflicts of interest.

## Data Availability

The data that support the findings of this study are openly available in Christine Kerubo Onyoni at https://wiley.atyponrex.com/submission/dashboard (Reference Number 6345685).
